# Protection of Visual Functions by Human Neural Progenitors in a Rat Model of Retinal Disease

**DOI:** 10.1371/journal.pone.0000338

**Published:** 2007-03-28

**Authors:** David M. Gamm, Shaomei Wang, Bin Lu, Sergei Girman, Toby Holmes, Nicholas Bischoff, Rebecca L. Shearer, Yves Sauvé, Elizabeth Capowski, Clive N. Svendsen, Raymond D. Lund

**Affiliations:** 1 Department of Ophthalmology and Visual Sciences, University of Wisconsin, Madison, Wisconsin, United States of America; 2 Departments of Anatomy and Neurology, University of Wisconsin, Madison, Wisconsin, United States of America; 3 Waisman Center Stem Cell Research Program, University of Wisconsin, Madison, Wisconsin, United States of America; 4 Department of Ophthalmology and Visual Sciences, Moran Eye Center, University of Utah, Salt Lake City, Utah, United States of America; 5 Department of Ophthalmology, Casey Eye Institute, Oregon Health and Sciences University, Portland, Oregon, United States of America; 6 Department of Ophthalmology, University of Alberta, Edmonton, Alberta, Canada; University of Washington (Seattle), United States of America

## Abstract

**Background:**

A promising clinical application for stem and progenitor cell transplantation is in rescue therapy for degenerative diseases. This strategy seeks to preserve rather than restore host tissue function by taking advantage of unique properties often displayed by these versatile cells. In studies using different neurodegenerative disease models, transplanted human neural progenitor cells (hNPC) protected dying host neurons within both the brain and spinal cord. Based on these reports, we explored the potential of hNPC transplantation to rescue visual function in an animal model of retinal degeneration, the Royal College of Surgeons rat.

**Methodology/Principal Findings:**

Animals received unilateral subretinal injections of hNPC or medium alone at an age preceding major photoreceptor loss. Principal outcomes were quantified using electroretinography, visual acuity measurements and luminance threshold recordings from the superior colliculus. At 90–100 days postnatal, a time point when untreated rats exhibit little or no retinal or visual function, hNPC-treated eyes retained substantial retinal electrical activity and visual field with near-normal visual acuity. Functional efficacy was further enhanced when hNPC were genetically engineered to secrete glial cell line-derived neurotrophic factor. Histological examination at 150 days postnatal showed hNPC had formed a nearly continuous pigmented layer between the neural retina and retinal pigment epithelium, as well as distributed within the inner retina. A concomitant preservation of host cone photoreceptors was also observed.

**Conclusions/Significance:**

Wild type and genetically modified human neural progenitor cells survive for prolonged periods, migrate extensively, secrete growth factors and rescue visual functions following subretinal transplantation in the Royal College of Surgeons rat. These results underscore the potential therapeutic utility of hNPC in the treatment of retinal degenerative diseases and suggest potential mechanisms underlying their effect *in vivo*.

## Introduction

Retinal degenerative diseases that target photoreceptors or the adjacent retinal pigment epithelium (RPE) affect millions of people worldwide. Similar to many other neurodegenerative diseases, no effective treatments are available for patients afflicted with these blinding disorders. With advances in stem and progenitor cell technology, however, novel cell-based therapies are being envisioned [1]–[4].

In the CNS, transplanted human neural progenitor cells derived from prenatal cortex (hNPC^ctx^) display characteristics important for cell-based rescue of degenerating neurons. They are highly expandable in culture [5], demonstrate a capacity to survive, migrate and integrate into damaged neural tissue [6]–[9], and can delay cell death and/or functional loss in multiple animal models of neurodegenerative disease [10]–[12]. Furthermore, they can express transgenes encoding specific neurotrophic factors that have protective effects on neighboring host neurons [11], [12]. Despite their promise, the utility of hNPC^ctx^ to rescue vision following subretinal transplantation in models of retinal degenerative disease has not been examined to our knowledge. Such a treatment approach might reduce the need for tailored gene replacement strategies for the genetically heterogenous group of disorders collectively referred to as retinitis pigmentosa. It may also be applicable in genetically complex or multifactorial retinal degenerative diseases such as age-related macular degeneration and glaucoma.

In the past, neural stem and progenitor cells from various sources were introduced into eyes with the thought that they might differentiate and replace photoreceptors lost in retinal disease [13]–[19]. Reports showed that while neural progenitors migrated into the retina and assumed the morphology of neurons, they failed to express retina-specific markers, including those characteristic of photoreceptors. However, their impact on host retinal function was not tested. The aim of the present study was to determine whether unmodified hNPC^ctx^ could rescue visual functions and if engineered expression of a neurotrophic factor could enhance such effects. Glial cell line-derived neurotrophic factor (GDNF) was selected based on clear evidence that it increases neuronal sprouting, prevents cell death [20], [21] and has neuroprotective effects in the brain [11], [22]–[24], spinal cord [12] and retina [25]–[30], and because receptors for GDNF are expressed within mature retina [26], [31]–[34].

We transplanted unmodified and GDNF-expressing hNPC^ctx^ (hNPC^ctx^-GDNF) to the subretinal space of the Royal College of Surgeons (RCS) rat. In this well-studied model of autosomal recessive retinitis pigmentosa and secondary photoreceptor degeneration, a MERTK mutation in the RPE compromises their ability to phagocytose shed photoreceptor outer segments [35]–[37]. This defect produces a debris zone between photoreceptors and RPE, with subsequent loss of the photoreceptors themselves. We found that unmodified hNPC^ctx^ possess a striking ability to preserve retinal activity and sustain a wide range of visual functions. Some, but not all, of these effects were augmented in the presence of GDNF-expressing cells. This report is significant for the unique disposition of the transplanted hNPC^ctx^ and for the subsequent levels of functional rescue achieved, which are among the best encountered in the RCS rat [29], [38]–[43].

## Methods

### Cell culture

Human NPC^ctx^ were isolated from post mortem fetal cortical brain tissue at 13.5 weeks gestation and designated as cell culture M031. The method of collection conformed to the NIH guidelines for the collection of such tissues, as well as the IRB requirements for the University of Wisconsin. Human NPC^ctx^ were cultured as spherical aggregates (neurospheres) in DMEM/HAMS F12 (3∶1) supplemented with B27 (1∶50; Gibco, Carlsbad, CA), 20 ng/ml EGF (Sigma-Aldrich, St. Louis, MO), 20 ng/ml FGF-2 (R&D Systems, Minneapolis, MN) and 5 µg/ml heparin (Sigma). Neurospheres were passaged by chopping as described previously [5] and half the medium was exchanged every four days. After four weeks in culture, the FGF-2, heparin and B27 were removed and N2 (1∶100; Invitrogen) was added. After ten weeks in culture, 10 ng/ml leukemia inhibitory factor (Chemicon, Temecula, CA) was also added.

### Lentiviral infection

A self-inactivating lentiviral construct containing a mouse phosphoglycerate kinase-1 internal promoter driving the human gene encoding GDNF [44] was used to generate GDNF-secreting hNPC^ctx^. Prior to infection, high-titer lentiviral stocks were obtained by ultracentrifugation and the particle content of individual batches was determined by p24 antigen ELISA and RT-qPCR quantification of viral RNA [45]. Human NPC^ctx^ neurospheres were incubated with Accutase (Chemicon; one ml per estimate of 10 million cells) for 10 minutes, followed by a five minute incubation with an equal volume of 0.2% trypsin inhibitor. After removal of the trypsin inhibitor, the spheres were washed three times with 10 ml of medium. Cells were dissociated by trituration, counted on a hemocytometer and resuspended in conditioned medium at 1000 cells/µl. 300,000 cells were plated per well of a 24-well plate (minimum 10 wells) and mixed with virus (80 ng p24/10^6^ cells) diluted in 100 µl of fresh medium. Cells re-associated in the presence of virus and formed spheres within three days, whereupon they were collected and seeded in flasks at a density of approximately 500,000 cells/ml. After one month, expanded cultures of transgenic (hNPC^ctx^-GDNF) and unmodified (hNPC^ctx^) neurospheres from the same original culture were cryoprotected and banked in liquid nitrogen for later use. For the transplantation experiments performed in this study, hNPC^ctx^-GDNF and hNPC^ctx^ neurosphere cultures were thawed and further expanded to passage 24 (equivalent to approximately 39 population doublings), a point at which they exhibit steady-state growth and cell fate potential *in vitro* [46].

### Preparation of cells for transplantation

One hour before surgery, hNPC^ctx^-GDNF or hNPC^ctx^ neurospheres were dissociated for 10 minutes in Accutase (1 ml/10 million cells) followed by inactivation with an equal volume of 0.2% trypsin inhibitor. Neurospheres were washed twice with 10 ml of medium, gently triturated, and counted on a hemocytometer. Cell suspensions were diluted to a final concentration of 10^4^ cells/µl in DMEM/F12 (3∶1) and kept on ice until transplantation. Trypan blue dye exclusion was performed on cell suspensions prior to and immediately following each transplantation session, which showed greater than 90% and 75% cell survival, respectively.

### Growth factor ELISAs and GDNF immunocytochemistry

Neurospheres containing either hNPC^ctx^ or hNPC^ctx^-GDNF were dissociated with Accutase, washed and resuspended in plating medium (DMEM/F12 (3∶1) with 2% B27) at a density of 1000 cells/µl. Cells were plated either on glass coverslips (40,000 cells/coverslip) coated with poly-L lysine and laminin or six-well plates (10^6^ cells/well) coated with laminin alone. Cells were then maintained for three weeks by exchanging half the media with fresh plating media every three to four days. After three weeks, all medium from the six-well cultures was removed, followed by a single media wash and replacement with fresh medium for 24 hours. Conditioned medium was collected and protein levels of GDNF, IGF-1, and FGF-2 were quantified by ELISA (R&D Systems) according to the manufacturer's protocols. The plated cells were then dissociated and counted using a hemocytometer in order to express results as picograms or nanograms of growth factor produced per day per million cells. Coverslips plated with acutely dissociated hNPC^ctx^-GDNF or hNPC^ctx^ were fixed with 4% paraformaldehyde, washed with PBS, blocked in 5% normal donkey serum and 0.1% Triton X-100, and incubated with goat anti-GDNF (1∶100; R&D Systems) primary antibody followed by donkey anti-goat Cy3-conjugated secondary antibody (1∶1000; Jackson IR). Nuclei were counterstained with Hoechst 33258 (1∶10,000; Sigma) and coverslips were mounted in GelTol Aqueous mounting media (Immunotech). At least five fields from each of three coverslips were photographed with a Nikon E600 equipped with epifluorescence, using SPOTcam and SPOT advanced software (Diagnostic Instruments, Inc.). Fluorescence was quantified using Metamorph software and data was expressed as mean±SEM.

### Animals

Twenty-one day old pigmented dystrophic RCS rats (*rdy^+^, p^+^)* received unilateral subretinal injections of hNPC^ctx^ (2×10^4^/2 µl/eye) (*n* = 21), hNPC^ctx^-GDNF (*n* = 11), or carrier medium only (*n* = 4) (sham surgery). Further sham-operated RCS rats from separate, concurrent studies [42], [43] were available for comparison and yielded similar results (these animals were not included in the present study). For each animal included in this study, fellow eyes served as untreated, internal controls. All animals were maintained on cyclosporine A (Novartis), administered in the drinking water (210 mg/l; resulting blood concentration of around 300 µg/liter [40]), from one day prior to transplantation until they were sacrificed. All animals also received daily dexamethasone injections (1.6 mg/kg, i.p.) for 2 weeks, starting from the day of transplantation. The studies were conducted with approval and under the supervision of the Institutional Animal Care Committee at the University of Utah; all animals were treated in accordance with the Policies on the Use of Animals and Humans in Neuroscience Research, approved by the Society for Neuroscience in January 1995.

### Transplantation

In order to study rescue effects following surgery, donor cells were introduced at P21, an age preceding major onset of photoreceptor loss. Separate transplantation sessions were performed using different batches of cells in order to ensure that results were repeatable. Suspensions of hNPC^ctx^ or hNPC^ctx^-GDNF containing about 2×10^4^ cells were delivered into the subretinal space of one eye through a small scleral incision as a suspension in 2 µl of DMEM/F12 medium (Invitrogen) using a fine glass pipette (internal diameter 75–150 µm) attached by tubing to a 10 µl Hamilton syringe. The cornea was punctured to reduce intraocular pressure and limit the efflux of cells. A sham-operated group was treated in an identical manner, except carrier medium alone was injected. Immediately after injection, the fundus was examined for retinal damage or signs of vascular distress. Any animal showing such problems was removed from the study and not included in the final animal counts.

### Electroretinogram (ERG)

Dark adapted full field ERG responses were recorded at approximately P100 as in previous studies [38]. Cone responses were isolated by employing a double flash protocol in which a conditioning flash was followed by a probe flash one second later. The conditioning flash served to transiently saturate rods so that they were rendered unresponsive to the probe flash. The intensity of the conditioning flash for complete rod bleaching was set to 1.4 log cds/m^2^ for all tests. A composite b-wave was obtained by presenting the probe flash alone, *i.e.*, without being preceded by a conditioning flash. The response to the probe flash (1.4 log cds/m^2^), preceded by the conditioning flash, was taken as reflecting cone-driven activity, and allowed the rod contribution to be derived by subtraction of the cone response from the composite response. Special care was taken to maintain the electrode placement in a consistent position in all animals. Averages of 3–5 traces (set 2 minutes apart to ensure recovery of rod responsiveness) were obtained.

### Visual acuity records obtained by measuring optomotor responses

Animals were tested for spatial visual acuity at approximately P100 using an Optomotry testing apparatus [47]. This device consists of a rotating cylinder covered with a vertical sine wave grating presented in virtual three-dimensional (3-D) space on four computer monitors arranged in a square. Unrestrained rats were placed on a platform in the center of the square, where they tracked the grating with reflexive head movements. The spatial frequency of the grating was clamped at the viewing position by repeatedly re-centering the ‘cylinder’ on the head of the test subject. Acuity was quantified by increasing the spatial frequency of the grating using a psychophysics staircase progression until the optokinetic reflex was lost, thereby obtaining a maximum threshold.

### Luminance threshold responses

To measure luminance threshold, single and multiunit activity was recorded in the superior colliculus (SC) at approximately P100 using a modification of a previously described procedure [48]. Recordings were made from the superficial layers of the SC to a depth of 100–300 µm using glass-coated tungsten electrodes (resistance: 0.5 MΩ; bandpass 500 Hz–5 KHz). Brightness of a 5° spot was varied with neutral density filters (minimum steps of 0.1 log) until a response was obtained that was double the background activity, yielding the threshold level for that point on the visual field. A total of 15–20 positions were recorded from each side of the SC, which provided a map of light sensitivity across the SC. Data was expressed in table form as mean percentage of SC area possessing a luminance threshold below a particular level.

### Histology

At the end of testing at P100 or P150, rats were euthanized with sodium pentobarbital (Sigma) overdose and perfused with phosphate buffered saline (PBS). The superior pole of each eye was marked with a suture to maintain orientation. The eyes were then removed, immersed in 2% paraformaldehyde for one hour, infiltrated with sucrose, embedded in OCT and cut into 10 µm horizontal sections on a cryostat. Four sections (50 µm apart) were collected per slide as 5 series. One series was stained with cresyl violet (CV) for assessing the injection site and retinal lamination. The remaining slides were used for antibody staining, following previous protocols [49]. The antibodies used in this study are listed in Table 1. Retinal sections were examined by regular and confocal microscopy. Some blocks were embedded in plastic and semi-thin sections were collected for examination at higher resolution.

**Table 1:** Sources and Working Dilutions of Antibodies for Immunohistochemistry.

Antigen	Antiserum	Working dilution	Source
Human nestin	Mouse anti-nestin	1∶200	Chemicon International
Human nuclei	Mouse anti-human nuclei	1∶300	Chemicon International
Human PCNA	Mouse anti-PCNA	1∶3000	Sigma
Cone arrestin	Rabbit anti-LUMIJ	1∶500	Drs. Zhu and Craft, U. So. California
PKCα	Rabbit anti-PKCα	1∶1000	Sigma
Rhodopsin	Mouse anti-rhodopsin	1∶1000	Dr. Molday, U British Columbia
Calbindin	Rabbit anti-calbindin	1∶1000	Swant, Switzerland
Parvalbumin	Rabbit anti-parvalbumin	1∶3000	Swant, Switzerland
Bestrophin	Mouse anti-bestrophin	1∶100	Chemicon International
RPE65	Mouse anti-RPE65	1∶300	Chemicon International

### Data Analysis

Statistical analyses were performed using GraphPad Prism version 3.02 for Windows (GraphPad Software, San Diego California USA). Data are presented as mean±standard error of the mean (SEM). Statistical analyses were made using either Student's two-tailed unpaired *t* test or analysis of variance (ANOVA) as specified in the figure legends, and Newman-Keuls procedure was used for multiple comparison analysis. Differences were considered to be significant at *p*<0.05.

## Results

### Human neural progenitors have an innate capacity to secrete specific growth factors and can be genetically modified to release GDNF

Prior to transplantation, we analyzed the *in vitro* production of specific growth factors by unmodified hNPC^ctx^ and by hNPC^ctx^ transduced with a lentiviral gene construct designed to constitutively express GDNF (hNPC^ctx^-GDNF) [11], [44]. This provided an *a priori* indication of their potential to influence host cells in a paracrine manner, a mechanism postulated to underlie protective effects observed in previous cell transplant studies [29], [39], [42], [43].

**Figure 1:**
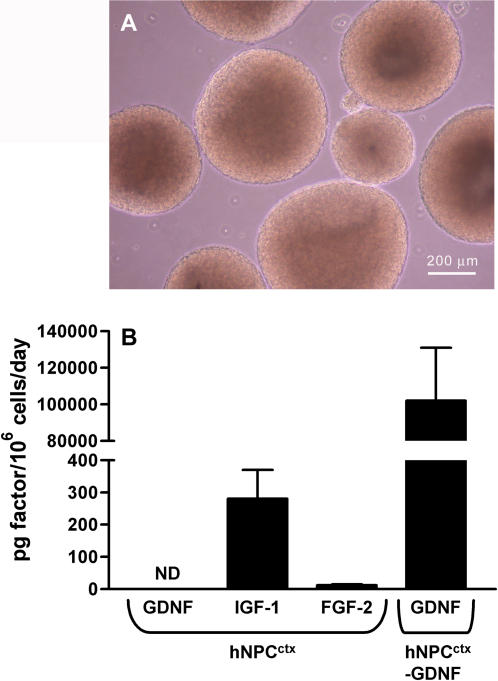
*Unmodified and transgenic human neural progenitor cells secrete neuroprotective factors in vitro.* (A) Light microscopic appearance of neurospheres cultured from human prenatal cortical progenitor cells (hNPC^ctx^). Transgenic neurospheres formed following infection of dissociated hNPC^ctx^ with a lentiviral vector encoding GDNF had an identical appearance in culture. (B) ELISA results quantifying the release of GDNF, IGF-1 and FGF-2 into conditioned media from hNPC^ctx^ and hNPC^ctx^–GDNF cultures. IGF-1 and FGF-2 were secreted by hNPC^ctx^, while GDNF was only detectable in transgenic hNPC^ctx^-GDNF cultures. Data are expressed as mean±SEM from *n* = 3–6 independent samples. (ND: not detectable).

ELISA was used to quantify the release of three molecules with potential neuroprotective activity in the brain or retina: IGF-1 [50]–[52], FGF-2 [26], [53], [54] and GDNF [11], [22]–[30]. Human NPC^ctx^ or NPC^ctx^-GDNF were grown as neurospheres for approximately 39 population doublings (Figure 1A), at which time they were dissociated and analyzed for growth factor release. IGF-1 and FGF-2 were secreted into hNPC^ctx^ conditioned medium at a rate of 280±90 and 12.0±3.0 picograms/10^6^ cells/day, respectively (Figure 1B). Unmodified hNPC^ctx^ did not secrete GDNF at levels above background. In contrast, transgenic hNPC^ctx^-GDNF cultures released GDNF at a rate of 102.5±29.9 nanograms/10^6^ cells/day (Figure 1B). Immunocytochemical analysis revealed that 75.9±11.8% of the hNPC^ctx^–GDNF population stably expressed the lentiviral gene construct. Thus, hNPC^ctx^ have the capacity to secrete endogenous growth factors of known importance for the maintenance and survival of retinal neurons, as well as the ability to constitutively express transgenes encoding selected neurotrophins.

### Subretinal injection of human neural progenitors preserves retinal and visual functions

**Figure 2:**
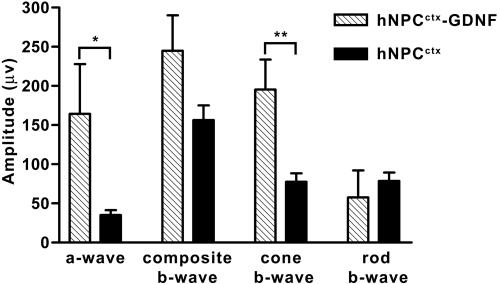
*The capacity of human neural progenitors to preserve retinal function is augmented by GDNF expression.* ERG response amplitudes to full field light stimulation were recorded at approximately P100 in RCS rats injected with hNPC^ctx^–GDNF (*n* = 9), hNPC^ctx^ (*n* = 21) or medium alone (*n* = 3) into the subretinal space. Individual components of the ERG waveform (a-wave, composite b-wave, cone b-wave and rod b-wave) reveal relative contributions of different retinal cells to the overall functional activity of the retina. Cone b-waves were delineated by a double flash protocol and the rod-attributable b-wave was derived by subtraction from the composite level. Eyes injected with hNPC^ctx^–GDNF demonstrated significantly greater a-wave and cone b-wave amplitudes than those receiving hNPC^ctx^ (*p<0.05; **p<0.01; Student's unpaired *t*-test). In contrast, the composite b-wave and rod b-wave amplitudes were not statistically different between the two groups. Control eyes injected with medium alone (sham-operated eyes) had no recordable ERG waves and are not included in the graph. Data are expressed as mean ± SEM.

RCS rats received unilateral subretinal injections of hNPC^ctx^, hNPC^ctx^-GDNF or medium alone (sham) at P21. Fellow, untreated eyes served as internal controls for each animal. The first test performed was ERG, which provides a gross measure of retinal function and an indication of relative rod and cone efficacy. In the scotopic-adapted RCS rat, the ERG a-wave (indicative mainly of rod activity) disappears by P60, while the composite b-wave (comprising rod and cone activity) is largely lost around P100 [39]. At approximately P100, eyes receiving either hNPC^ctx^ (*n* = 21) or hNPC^ctx^-GDNF (*n* = 9) injections retained robust ERG responses (Figure 2). In contrast, sham-treated eyes (*n* = 3) had no measurable ERG responses at this age. Further comparison of eyes injected with hNPC^ctx^-GDNF or hNPC^ctx^ revealed significantly greater a-wave and cone b-wave amplitudes in the GDNF-secreting group (a-wave: 164.3±63.7 µv vs. 35.2±6.2 µv (*p*<0.05); cone b-wave: 195.4±38.1 µv vs. 77.7±10.6 µv (*p*<0.01), respectively). For perspective, non-dystrophic rats yielded a-wave and cone b-wave responses of 279±172 µv and 357±183 µv, respectively. Thus, eyes grafted with hNPC^ctx^-GDNF retained ERG activity at approximately 58.8% (a-wave) and 54.6% (cone b-wave) of the level of normal, non-dystrophic animals. Composite b-wave and rod b-wave amplitudes were also well-preserved in the cell-injected eyes, but no significant difference was observed between the hNPC^ctx^-GDNF and hNPC^ctx^ groups (composite b-wave: 244.9±45.3 µv vs. 156.4±18.7 µv (*p* = 0.12); rod b-wave: 57.6±34.5 µv vs. 78.7±10.5 µv (*p* = 0.84), respectively).

**Figure 3:**
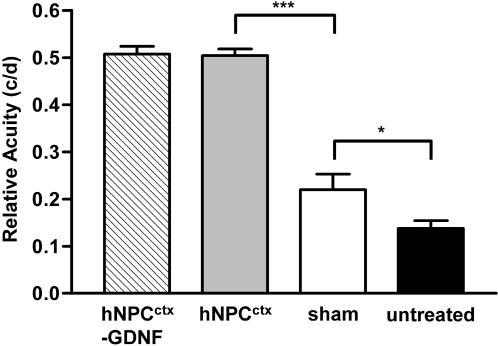
*Spatial visual acuity is preserved in eyes receiving human neural progenitor cell grafts.* Rapid, non-invasive measurements of spatial visual acuity thresholds were obtained using the OptoMotry head tracking apparatus. At approximately P100, eyes receiving subretinal hNPC^ctx^-GDNF (*n* = 11) or hNPC^ctx^ (*n* = 21) demonstrated superior spatial visual acuity compared to sham-injected eyes (*n* = 4; ***p<0.001), with best-grafted animals yielding near-normal responses (0.6 c/d). Spatial visual acuity was also significantly better in sham-treated vs. untreated (*n* = 29; *p<0.05) eyes, reflecting the known “sham effect.” Statistical significance was determined via one-way ANOVA with the Newman-Keuls procedure for multiple comparison analysis.

We next asked whether spatial visual acuity was affected by the transplants using the optomotor, or head tracking, test [47], [55]. At approximately P100, eyes receiving hNPC^ctx^ (*n* = 21) injections performed significantly better than sham-operated (*n* = 4) or untreated (*n* = 29) control eyes (0.50±0.01 c/d vs. 0.22±0.03 c/d (*p*<0.001) or 0.14±0.02 c/d (*p*<0.001), respectively) (Figure 3). Eyes injected with hNPC^ctx^-GDNF (*n* = 11) possessed an average visual acuity similar to hNPC^ctx^ recipients (0.51±0.02 c/d vs. 0.50±0.01 c/d, *p* = 0.90), with some animals in both groups retaining acuities within the normal, non-dystrophic range (0.56–0.62 c/d). Sham-operated eyes also retained significantly better spatial visual acuity than untreated eyes (0.22±0.03 c/d vs. 0.14±0.02 c/d, *p*<0.05), as shown previously. However, cell-grafted animals tested at P150 continued to perform as high as 0.49 c/d (data not shown), whereas no measurable response was observed in any of the sham-operated or untreated retinas at this late time point. Of note, the sham responses obtained in these experiments were essentially identical to those obtained in other studies using the RCS rat [43].

**Figure 4:**
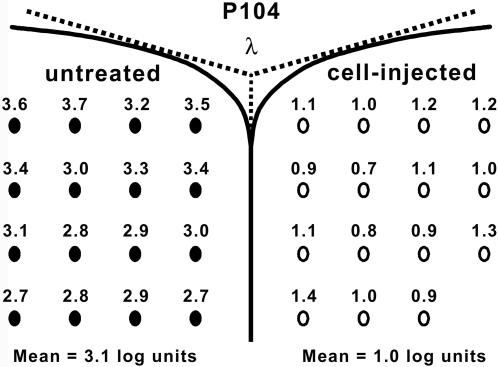
*Visual field is preserved in eyes receiving human neural progenitor cell grafts.* Luminance threshold responses were recorded at approximately P100 from multiple points within the superior colliculus (SC) contralateral to the eye being tested. This method quantifies functional sensitivity to light across the visual field of an eye. The topographical map depicts the luminance threshold responses (measured in log units relative to background illumination of 0.02 cd/m^2^) at 15 points within the SC opposite the cell-injected eye of a best-performing animal at P104 (*right side* of figure). SC recordings (16 points) opposite the fellow, untreated eye (*left side* of figure) served as an internal control. Both hNPC^ctx^–GDNF and hNPC^ctx^ transplant recipients were included in the cell-injected group, which consisted of a select population of eyes that displayed superior performance on spatial visual acuity testing. Recordings falling at or below a threshold of 2.0 log units are indicated with *unfilled ovals*, while recordings above 2.0 are demarcated with *filled ovals*.

Luminance threshold recordings from the SC measure functional sensitivity across the visual field, which in turn provides a geographic indication of the magnitude and area of photoreceptor rescue across the retina [56]. In dystrophic RCS rats, threshold levels at P100 are greater than 3.0 log units above the background level of 0.02 log candela/m^2^. This is in comparison to non-dystrophic rats, which possess threshold levels less than 0.6 log units above background [48]. For the present study, recordings were made in a combined set of cell-injected animals who received either hNPC^ctx^ or hNPC^ctx^-GDNF. Eyes were specifically chosen from either group based on their superior performance on optomotor testing; therefore, comparisons between the hNPC^ctx^ and hNPC^ctx^-GDNF groups are not appropriate. Overall, cell-injected eyes (*n* = 10) performed significantly better than untreated eyes (*n* = 5) or those receiving sham injections (*n* = 3) (Figure 4 and Table 2). Specifically, 8.0±5.8% of the SC area of cell-injected eyes produced thresholds less than 0.8 log units, 22.0±8.5% produced thresholds less than 1.5 log units and 67.7±10.0% yielded thresholds less than 2.1 log units, with best test points falling within the normal, non-dystrophic range. These results are in contrast to sham-injected eyes, where only 14.8±8.3% of the SC area yielded thresholds below 2.1 log units.

**Table 2:** Summary of Visual Threshold Measurements from the Superior Colliculus in Different Groups of Animals.

Group	*n*	Mean area ± SEM of superior colliculus (in %) with log thresholds at level less than:							
		0.2	0.8	1.5	2.1	2.8	3.4	4.1	4.7
Cell-injected	10	0	8.0±5.8	22.0±8.5	67.7±10	83.8±9.6	96.7±3.3	98.3±1.7	99.2±0.8
Sham	3	0	0	0	14.8±8.3	66.3±6.4	97.6±2.4	100	100
Untreated	5	0	0	0	0	0	40.9±13.8	92.9±2.4	98.3±0.9

In summary, eyes receiving human neural progenitor cells retained dramatically better retinal and visual functions compared to control eyes at P100. Furthermore, some components of the ERG were augmented when hNPC^ctx^ were engineered to release GDNF.

### Transplanted human neural progenitors survive, integrate, and form a new pigmented subretinal cell layer that protects photoreceptors from degeneration

**Figure 5:**
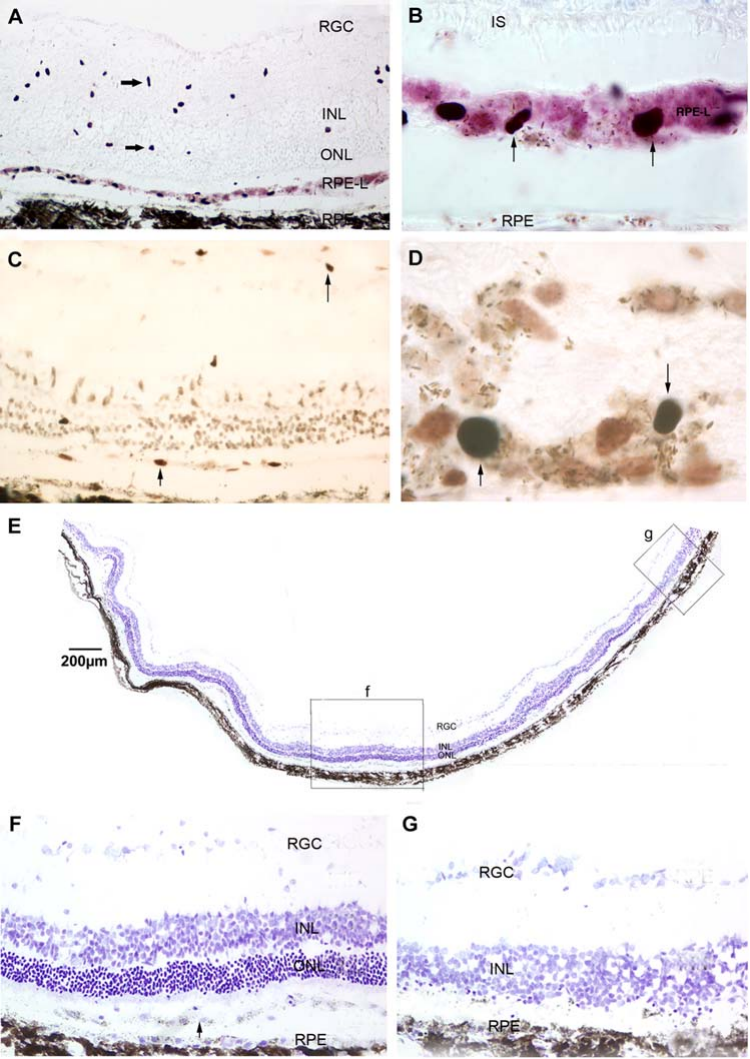
*Human neural progenitors survive as two distinct populations within the retina and promote photoreceptor rescue.* (A) Retinal section obtained from a P150 eye immunostained with human-specific nuclear marker. There is a pigmented RPE-like (RPE-L) layer of donor cells above the host RPE layer, whereas donor cells in the inner retina do not have pigment granules (*right-pointing arrows*). (B) High power image showing pigment granule-containing donor cells in the RPE-L region that are positive for human nuclear marker (*arrows*). Photoreceptor inner segments (IS) are visible above the pigmented donor cell layer, demonstrating partial preservation of photoreceptor structure. (C) Retinal section from the same eye used in panel A stained with proliferating cell nuclear antigen (PCNA) revealing occasional dividing cells in the RPE-L layer and inner retina at P150 (*arrows*). (D) High power image showing PCNA-positive cells in the RPE-L layer (*arrows*). (E) Low power view of a retina section obtained from the same eye used in panel A showing extensive rescue of photoreceptors within the outer nuclear layer (ONL) after subretinal injection of hNPC^ctx^–GDNF. The *boxes* labeled *f* and *g* correspond to the high power images depicted in panels F and G, respectively. (F) High power view of *box f* from panel E showing rescued ONL and the underlying, semi-continuous layer of donor cells between the photoreceptors and RPE (*arrow*). (G) High power view of *box g* from panel E showing non-rescued ONL distant from surviving subretinal donor cells. INL: inner nuclear layer; IS: inner segments; ONL: outer nuclear layer; RGC: retinal ganglion cell layer; RPE-L: RPE-like layer.

An antibody recognizing human-specific nuclear antigen was used to identify surviving hNPC^ctx^ and hNPC^ctx^-GDNF at P100 and P150. Both unmodified and genetically modified groups were found to have cells that migrated and survived in two distinct locations: (i) as a separate, nearly continuous, subretinal layer lying between the host RPE and photoreceptors, and (ii) as individual cells distributed throughout the neurosensory retina, especially within the inner retinal layers (Figure 5A).

Donor cells comprising the semi-continuous subretinal layer possessed intracellular pigment granules (confirmed on semi-thin sections) similar to host RPE cells, unlike those that migrated within the neurosensory retina, which remained unpigmented (Figure 5B). The pigmented subretinal donor cells failed to express two characteristic RPE markers, RPE65 [57] and bestrophin [58], arguing against the possibility that they had undergone full transdifferentiation. However, the photoreceptor outer segment debris zone normally found in the subretinal space of the RCS rat was nearly absent. A small number of donor cells in both the intraretinal and subretinal locations were immunopositive for proliferating cell nuclear antigen (PCNA) (Figure 5C and D), even in the oldest rats examined (P150). Despite this potential indication of continued cell division, there was no evidence of uncontrolled growth or tumor formation at any time, suggesting that donor cell proliferation might be regulated or balanced by cell death.

Qualitative examination of the host anatomical response to the presence of hNPC^ctx^ or hNPC^ctx^-GDNF revealed substantial preservation of the photoreceptor outer nuclear layer (ONL) overlying all subretinal donor cells (Figure 5E and F), with photoreceptor rescue gradually declining outside the distribution of the transplanted cells (Figure 5E and G). Distant from the subretinal grafts, the ONL was reduced to a single layer at P100 and discontinuous, scattered cells at P150 (Figure 5G), similar to untreated and sham-treated dystrophic retinas. Of interest, no ONL was seen in areas where donor cells were present exclusively in the inner retina, whereas a prominent ONL was present in areas where donor cells existed solely in the subretinal space. This observation suggests that subretinal localization of hNPC^ctx^ is necessary and sufficient to promote anatomic rescue of the ONL in this model.

**Figure 6:**
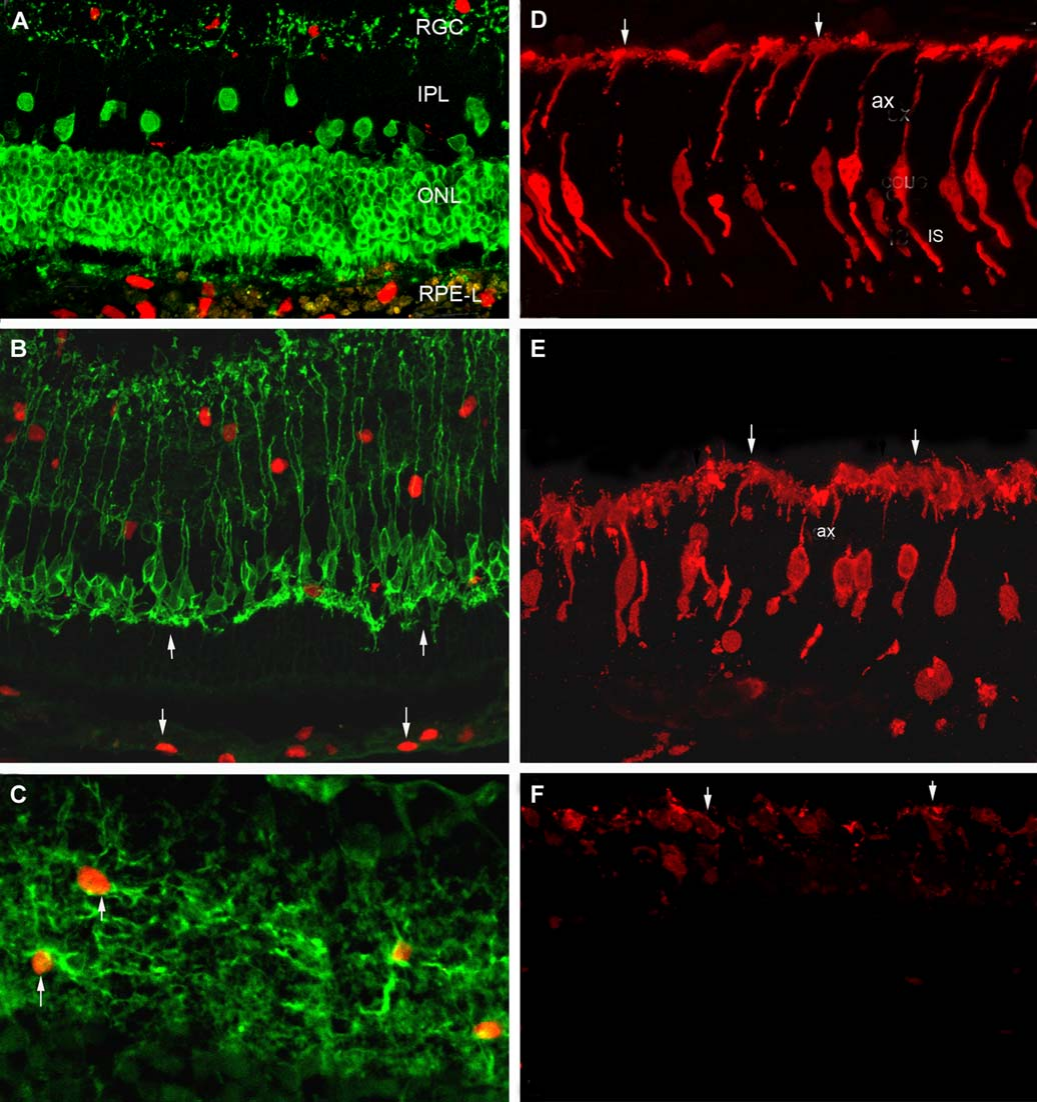
*Transplanted human neural progenitors express nestin in vivo and partially preserve host cone structure.* Confocal images of P150 dystrophic retina transplanted with hNPC^ctx^–GDNF and double stained with antibodies against human nuclear antigen (*red*) and either (A) recoverin, a photoreceptor and cone bipolar cell marker (*green*), or (B) protein kinase Cα (PKCα), a bipolar cell marker (*green*). *Down arrows* point to subretinal donor cell nuclei in panel B, while *up arrows* point to preserved dendrites of host rod bipolar cells. Note the location of donor cell nuclei in both the inner retina and subretinal space. (C) Confocal image of P150 dystrophic inner retina transplanted with hNPC^ctx^–GDNF and double stained with antibodies against nestin, a neural stem and progenitor cell marker (*green*) and human nuclear antigen (*red*). (D) Confocal image of non-dystrophic, control retina stained with cone arrestin antibody (*red*) showing a normal cone photoreceptor profile (IS: inner segments; ax: axon; *arrows* point to cone pedicles). (E) P150 dystrophic retina transplanted with hNPC^ctx^–GDNF and stained with cone arrestin antibody showing morphology of rescued cone photoreceptors (*arrows* point to cone pedicles). (F) Sham-operated retina at P150 stained with cone arrestin antibody (*arrows* point to rare remaining cone cell bodies). Results at P150 from retina transplanted with hNPC^ctx^ were similar to those receiving hNPC^ctx^–GDNF. IPL: inner plexiform layer; ONL: outer nuclear layer; RGC: retinal ganglion cell layer; RPE-L: RPE-like layer.

Donor cells (hNPC^ctx^ or hNPC^ctx^-GDNF) that migrated within the neurosensory retina did not express the retinal markers recoverin (Figure 6A), PKCα (Figure 6B), rhodopsin, parvalbumin or calbindin (latter markers not shown). However, the morphology of the host inner retinal cells was well-preserved in the area of donor cell migration, as evident from the PKCα antibody staining, which labeled normal-appearing rod bipolar cell dendrites (upward arrows in Figure 6B). Both the intraretinal and subretinal donor cell populations were immunopositive for nestin, a neural stem and progenitor cell marker, using a human-specific antibody. In addition, confocal microscopy showed a small portion of the transplanted hNPC^ctx^ remained GFAP-positive (not shown). Confocal microscopy further demonstrated an extensive network of nestin-positive cellular processes emanating from the transplanted cells present within the neurosensory retina (Figure 6C), which was not observed in the subretinal hNPC^ctx^ population.

While preservation of the ONL is evidence for a neuroprotective role for hNPC^ctx^ and GDNF within the retina, maintenance of visual function at the level observed in this study suggests at least partial retention of photoreceptor structure necessary for visual processing, particularly that associated with cones. To examine host cone photoreceptors, we performed antibody staining for cone arrestin antigen. In the area of best rescue, clearly identifiable cones were present at a density of 40–46 cells/mm across two-thirds of the histological section, even at 150 days of age (130 days after transplantation). This cone density is within the range seen in normal rat retinae, although cone processes in the rescued regions were clearly shorter than normal and poorly organized (Figure 6D and E). In contrast, cone photoreceptors were essentially absent in sham-operated or untreated eyes at the same time point (Figure 6F). This finding correlates with the partial preservation of cone ERG activity, visual acuity and visual field observed in cell-transplanted eyes.

## Discussion

Results from this study show that neural progenitor cells derived from developing human cortex promote long-term preservation of vision after subretinal transplantation in the RCS rat. Using three independent tests performed at P90-100, hNPC^ctx^- and hNPC^ctx^-GDNF-transplanted eyes demonstrated retention of visual functions at levels among the best documented in the RCS rat model [29], [38], [40]–[43], [56], [59], [60]. These results correlated with survival of photoreceptors, including cone cells, which are required for optimal daylight vision. In contrast, sham injections of medium alone failed to achieve significant sustained functional rescue, consistent with previous studies. It has also been shown that fibroblasts [43], [60] and placenta-derived progenitor cells [43] are unable to maintain visual functions long-term, further indicating that the rescue effects seen in the RCS rat model are not the result of a non-specific event. Of note, while vision rescue requires preservation of some functional photoreceptors, the mere presence of photoreceptor cells in the ONL of transplanted RCS rats does not assure function [61]. Thus, we chose to emphasize quantitative functional responses to hNPC^ctx^ transplantation and support these findings with a more qualitative investigation of the host anatomical impact.

Histological examination reveals that hNPC^ctx^ survive, migrate and assume two substantially different appearances and distributions following transplantation into the subretinal space of RCS rats. These findings were seen regardless of whether the cells were previously transduced with a GDNF-expressing construct. One population is non-pigmented and diffusely distributed within the inner retina, while the other is a pigment granule-containing, RPE-like layer located between the host neurosensory retina and RPE. In previous studies using other donor cell types, significant intraretinal migration was not seen [39], [41], nor were pigment granules found in donor cells not originally derived from RPE [39]. However, similar to other reports introducing forebrain–derived stem cells into the retina [13]–[17], donor cells used in this study did not express markers typical of host retinal cells. Our observations imply that donor cell location within host tissue can influence their apparent phenotype, even though they lack critical markers of the cells they come to resemble.

The mechanism by which human neural progenitor cells exert their effects within the retina is not wholly clear, but is likely due in part to growth factor production [39] and possibly also to the manifestation of some RPE-like properties. With respect to factor production, we have detailed at least two factors, IGF-1 and FGF-2, that hNPC^ctx^ produce *in vitro* that could be effective in promoting vision and photoreceptor preservation. A more comprehensive survey may identify others. Thus, transplanted hNPC^ctx^ have the potential to release multiple growth factors, which may act synergistically to slow photoreceptor degeneration [62], [63]. The superior performance of hNPC^ctx^-GDNF is consistent with both the known role of GDNF as a neuroprotective molecule within the retina [25]–[30] and the established ability of hNPC^ctx^ to function as a cell-based drug delivery vector in diverse CNS tissues [11], [12]. The additional capacity of hNPC^ctx^ to migrate extensively within the subretinal space and inner retina allows them to deliver molecules of therapeutic interest not only for outer retinal disease (*e.g.*, retinitis pigmentosa and age-related macular degeneration), but inner retinal disorders as well (*e.g.*, glaucoma). Panretinal donor cell migration also affords better access to Müller glia, which bind and mediate host responses to some neurotrophic factors, including GDNF [26].

The additional question of whether hNPC^ctx^ might mimic some of the functions of RPE is an intriguing one. A population of these cells forms a layer deep to the photoreceptors, where they contain intracellular pigment granules and appear superficially like an extra RPE layer, even though they do not express at least two characteristic RPE proteins. The presence of intracellular pigment granules along with the absence of a subretinal cellular debris zone raise the possibility that these donor cells have (or acquire) the capacity to phagocytose surrounding waste material. As this is one function of healthy RPE [64], such activity may contribute to the cell transplant-mediated rescue observed, a possibility that is being explored further.

The fact that donor cells continue to divide until P150 is a matter of both concern and optimism. Previous work has shown that ES-derived RPE cells can develop teratomas [65], although not in all cases [42]. However, in the present study there is no evidence of untoward donor cell proliferation or tumor formation up to at least 130 days post-transplantation, suggesting that cell division is a regulated or balanced event. Indeed, persistent cell division may contribute to the sustained high performance of hNPC^ctx^ transplant recipients over time. Even so, later time points are needed to ensure that tumors never form within the retina after transplantation of hNPC^ctx^.

In summary, transplanted hNPC^ctx^ display a novel profile of properties that produce profound rescue of visual functions in the RCS rat, an animal model of photoreceptor loss secondary to RPE dysfunction. The potential for native or modified hNPC^ctx^ to deliver neurotrophins and rescue cones and photopic vision in primary rod degeneration models also needs to be assessed. However, current results point to a possible role for hNPC^ctx^ in the treatment of at least some forms of human retinal degenerative diseases and highlight the versatility and efficacy of these cells as therapeutic tools in a broad range of neurodegenerative disorders [10]–[12], [66]. A current clinical trial investigating the use of transplanted human neural stem cells in Batten disease [67] will address questions concerning the safety of this cell type and provide important background for contemplating their clinical application in retinal disease.

## References

[1] Gage FH (2000). Mammalian neural stem cells.. Science.

[2] McKay R (1997). Stem cells in the central nervous system.. Science.

[3] Schwartz PH (2006). The potential of stem cell therapies for neurological diseases.. Expert Rev Neurother.

[4] Svendsen CN, Langston JW (2004). Stem cells for Parkinson disease and ALS: replacement or protection?. Nat Med.

[5] Svendsen CN, ter Borg MG, Armstrong RJ, Rosser AE, Chandran S (1998). A new method for the rapid and long term growth of human neural precursor cells.. J Neurosci Methods.

[6] Svendsen CN, Caldwell MA, Shen J, ter Borg MG, Rosser AE (1997). Long-term survival of human central nervous system progenitor cells transplanted into a rat model of Parkinson's disease.. Exp Neurol.

[7] Ostenfeld T, Caldwell MA, Prowse KR, Linskens MH, Jauniaux E, Svendsen CN (2000). Human neural precursor cells express low levels of telomerase in vitro and show diminishing cell proliferation with extensive axonal outgrowth following transplantation.. Exp Neurol.

[8] Fricker RA, Carpenter MK, Winkler C, Greco C, Gates MA, Bjorklund A (1999). Site-specific migration and neuronal differentiation of human neural progenitor cells after transplantation in the adult rat brain.. J Neurosci.

[9] Vescovi AL, Gritti A, Galli R, Parati EA (1999). Isolation and intracerebral grafting of nontransformed multipotential embryonic human CNS stem cells.. J Neurotrauma.

[10] McBride JL, Behrstock SP, Chen EY, Jakel RJ, Siegel I (2004). Human neural stem cell transplants improve motor function in a rat model of Huntington's disease.. J Comp Neurol.

[11] Behrstock S, Ebert A, McHugh J, Vosberg S, Moore J (2006). Human neural progenitors deliver glial cell line-derived neurotrophic factor to parkinsonian rodents and aged primates.. Gene Ther.

[12] Klein SM, Behrstock S, McHugh J, Hoffmann K, Wallace K (2005). GDNF delivery using human neural progenitor cells in a rat model of ALS.. Hum Gene Ther.

[13] Kurimoto Y, Shibuki H, Kaneko Y, Ichikawa M, Kurokawa T (2001). Transplantation of adult rat hippocampus-derived neural stem cells into retina injured by transient ischemia.. Neurosci Lett.

[14] Nishida A, Takahashi M, Tanihara H, Nakano I, Takahashi JB (2000). Incorporation and differentiation of hippocampus-derived neural stem cells transplanted in injured adult rat retina.. Invest Ophthalmol Vis Sci.

[15] Lu B, Kwan T, Kurimoto Y, Shatos M, Lund RD, Young MJ (2002). Transplantation of EGF-responsive neurospheres from GFP transgenic mice into the eyes of rd mice.. Brain Res.

[16] Mizumoto H, Mizumoto K, Shatos MA, Klassen H, Young MJ (2003). Retinal transplantation of neural progenitor cells derived from the brain of GFP transgenic mice.. Vision Res.

[17] Young MJ, Ray J, Whiteley SJ, Klassen H, Gage FH (2000). Neuronal differentiation and morphological integration of hippocampal progenitor cells transplanted to the retina of immature and mature dystrophic rats.. Mol Cell Neurosci.

[18] Takahashi M, Palmer TD, Takahashi J, Gage FH (1998). Widespread integration and survival of adult-derived neural progenitor cells in the developing optic retina.. Mol Cell Neurosci.

[19] Mizumoto H, Mizumoto K, Whiteley SJ, Shatos M, Klassen H, Young MJ (2001). Transplantation of human neural progenitor cells to the vitreous cavity of the Royal College of Surgeons rat.. Cell Transplant.

[20] Keller-Peck CR, Feng G, Sanes JR, Yan Q, Lichtman JW, Snider WD (2001). Glial cell line-derived neurotrophic factor administration in postnatal life results in motor unit enlargement and continuous synaptic remodeling at the neuromuscular junction.. J Neurosci.

[21] Blesch A, Tuszynski MH (2001). GDNF gene delivery to injured adult CNS motor neurons promotes axonal growth, expression of the trophic neuropeptide CGRP, and cellular protection.. J Comp Neurol.

[22] Lin LF, Doherty DH, Lile JD, Bektesh S, Collins F (1993). GDNF: a glial cell line-derived neurotrophic factor for midbrain dopaminergic neurons.. Science.

[23] Gash DM, Zhang Z, Ovadia A, Cass WA, Yi A (1996). Functional recovery in parkinsonian monkeys treated with GDNF.. Nature.

[24] Bjorklund A, Rosenblad C, Winkler C, Kirik D (1997). Studies on neuroprotective and regenerative effects of GDNF in a partial lesion model of Parkinson's disease.. Neurobiol Dis.

[25] McGee Sanftner LH, Abel H, Hauswirth WW, Flannery JG (2001). Glial cell line derived neurotrophic factor delays photoreceptor degeneration in a transgenic rat model of retinitis pigmentosa.. Mol Ther.

[26] Hauck SM, Kinkl N, Deeg CA, Swiatek-de Lange M, Schoffmann S, Ueffing M (2006). GDNF family ligands trigger indirect neuroprotective signaling in retinal glial cells.. Mol Cell Biol.

[27] Andrieu-Soler C, Aubert-Pouessel A, Doat M, Picaud S, Halhal M (2005). Intravitreous injection of PLGA microspheres encapsulating GDNF promotes the survival of photoreceptors in the rd1/rd1 mouse.. Mol Vis.

[28] Wu WC, Lai CC, Chen SL, Sun MH, Xiao X (2004). GDNF gene therapy attenuates retinal ischemic injuries in rats.. Mol Vis.

[29] Lawrence JM, Keegan DJ, Muir EM, Coffey PJ, Rogers JH (2004). Transplantation of Schwann cell line clones secreting GDNF or BDNF into the retinas of dystrophic Royal College of Surgeons rats.. Invest Ophthalmol Vis Sci.

[30] Frasson M, Picaud S, Leveillard T, Simonutti M, Mohand-Said S (1999). Glial cell line-derived neurotrophic factor induces histologic and functional protection of rod photoreceptors in the rd/rd mouse.. Invest Ophthalmol Vis Sci.

[31] Kretz A, Jacob AM, Tausch S, Straten G, Isenmann S (2006). Regulation of GDNF and its receptor components GFR-alpha1, -alpha2 and Ret during development and in the mature retino-collicular pathway.. Brain Res.

[32] Lindqvist N, Peinado-Ramonn P, Vidal-Sanz M, Hallbook F (2004). GDNF, Ret, GFRalpha1 and 2 in the adult rat retino-tectal system after optic nerve transection.. Exp Neurol.

[33] Jomary C, Darrow RM, Wong P, Organisciak DT, Jones S (2004). Expression of neurturin, glial cell line-derived neurotrophic factor, and their receptor components in light-induced retinal degeneration.. Invest Ophthalmol Vis Sci.

[34] Jing S, Wen D, Yu Y, Holst PL, Luo Y (1996). GDNF-induced activation of the ret protein tyrosine kinase is mediated by GDNFR-alpha, a novel receptor for GDNF.. Cell.

[35] D'Cruz PM, Yasumura D, Weir J, Matthes MT, Abderrahim H (2000). Mutation of the receptor tyrosine kinase gene Mertk in the retinal dystrophic RCS rat.. Hum Mol Genet.

[36] LaVail MM (2001). Legacy of the RCS rat: impact of a seminal study on retinal cell biology and retinal degenerative diseases.. Prog Brain Res.

[37] Feng W, Yasumura D, Matthes MT, LaVail MM, Vollrath D (2002). Mertk triggers uptake of photoreceptor outer segments during phagocytosis by cultured retinal pigment epithelial cells.. J Biol Chem.

[38] Sauvé Y, Pinilla I, Lund RD (2006). Partial preservation of rod and cone ERG function following subretinal injection of ARPE-19 cells in RCS rats.. Vision Res.

[39] Wang S, Lu B, Wood P, Lund RD (2005). Grafting of ARPE-19 and Schwann cells to the subretinal space in RCS rats.. Invest Ophthalmol Vis Sci.

[40] Coffey PJ, Girman S, Wang SM, Hetherington L, Keegan DJ (2002). Long-term preservation of cortically dependent visual function in RCS rats by transplantation.. Nat Neurosci.

[41] Lund RD, Adamson P, Sauvé Y, Keegan DJ, Girman SV (2001). Subretinal transplantation of genetically modified human cell lines attenuates loss of visual function in dystrophic rats.. Proc Natl Acad Sci USA.

[42] Lund RD, Wang S, Klimanskaya I, Holmes T, Ramos-Kelsey R (2006). Human embryonic stem cell-derived cells rescue visual function in dystrophic RCS rats.. Cloning Stem Cells.

[43] Lund RD, Wang S, Lu B, Girman S, Holmes T (2006). Cells isolated from umbilical cord tissue rescue photoreceptors and visual functions in a rodent model of retinal disease.. Stem Cells.

[44] Deglon N, Tseng JL, Bensadoun JC, Zurn AD, Arsenijevic Y (2000). Self-inactivating lentiviral vectors with enhanced transgene expression as potential gene transfer system in Parkinson's disease.. Hum Gene Ther.

[45] Capowski EE, Schneider BL, Ebert AD, Seehus CR, Szulc J (In press). Lentiviral vector-mediated genetic modification of human neural progenitor cells for ex vivo gene therapy.. J Neurosci Methods.

[46] Wright LS, Prowse KR, Wallace K, Linskens MH, Svendsen CN (2006). Human progenitor cells isolated from the developing cortex undergo decreased neurogenesis and eventual senescence following expansion in vitro.. Exp Cell Res.

[47] Prusky GT, Alam NM, Beekman S, Douglas RM (2004). Rapid quantification of adult and developing mouse spatial vision using a virtual optomotor system.. Invest Ophthalmol Vis Sci.

[48] Sauvé Y, Girman SV, Wang S, Lawrence JM, Lund RD (2001). Progressive visual sensitivity loss in the RCS rat: perimetric study in the superior colliculus and retinal anatomy.. Neurosci.

[49] Wang S, Lu B, Lund RD (2005). Morphological changes in the Royal College of Surgeons rat retina during photoreceptor degeneration and after cell-based therapy.. J Comp Neurol.

[50] Barber AJ, Nakamura M, Wolpert EB, Reiter CE, Seigel GM (2001). Insulin rescues retinal neurons from apoptosis by a phosphatidylinositol 3-kinase/Akt-mediated mechanism that reduces the activation of caspase-3.. J Biol Chem.

[51] Seigel GM, Chiu L, Paxhia A (2000). Inhibition of neuroretinal cell death by insulin-like growth factor-1 and its analogs.. Mol Vis.

[52] Zheng WH, Kar S, Dore S, Quirion R (2000). Insulin-like growth factor-1 (IGF-1): a neuroprotective trophic factor acting via the Akt kinase pathway.. J Neural Transm.

[53] Spencer B, Agarwala S, Gentry L, Brandt CR (2001). HSV-1 vector-delivered FGF2 to the retina is neuroprotective but does not preserve functional responses.. Mol Ther.

[54] Jin K, LaFevre-Bernt M, Sun Y, Chen S, Gafni J (2005). FGF-2 promotes neurogenesis and neuroprotection and prolongs survival in a transgenic mouse model of Huntington's disease.. Proc Natl Acad Sci USA.

[55] Douglas RM, Alam NM, Silver BD, McGill TJ, Tschetter WW, Prusky GT (2005). Independent visual threshold measurements in the two eyes of freely moving rats and mice using a virtual-reality optokinetic system.. Vis Neurosci.

[56] Sauve Y, Girman SV, Wang S, Keegan DJ, Lund RD (2002). Preservation of visual sensitivity recorded in the superior colliculus of RCS rats after retinal pigment epithelial cell transplantation.. Neurosci.

[57] Hamel CP, Tsilou E, Harris E, Pfeffer BA, Hooks JJ (1993). A developmentally regulated microsomal protein specific for the pigment epithelium of the vertebrate retina.. J Neurosci Res.

[58] Marmorstein AD, Marmorstein LY, Rayborn M, Wang X, Hollyfield JG, Petrukhin K (2000). Bestrophin, the product of the Best vitelliform macular dystrophy gene (VMD2), localizes to the basolateral plasma membrane of the retinal pigment epithelium.. Proc Natl Acad Sci USA.

[59] Girman SV, Wang S, Lund RD (2003). Cortical visual functions can be preserved by subretinal RPE cell grafting in RCS rats.. Vis Res.

[60] Lawrence JM, Sauve Y, Keegan DJ, Coffey PJ, Hetherington L (2000). Schwann cell grafting into the retina of the dystrophic RCS rat limits functional deterioration. Royal College of Surgeons.. Invest Ophthalmol Vis Sci.

[61] Girman SV, Wang S, Lund RD (2005). Time course of deterioration of rod and cone function in RCS rat and the effect of subretinal grafting; a light- and dark- adaptation study.. Vis Res.

[62] Caffe AR, Soderpalm AK, Holmqvist I, van Veen T (2001). A combination of CNTF and BDNF rescues rd photoreceptors but changes rod differentiation in the presence of RPE in retinal explants.. Invest Ophthalmol Vis Sci.

[63] Ogilvie JM, Speck JD, Lett JM (2000). Growth factors in combination, but not individually, rescue rd mouse photoreceptors in organ culture.. Exp Neurol.

[64] Strauss O (2005). The retinal pigment epithelium in visual function.. Physiol Rev.

[65] Arnhold S, Klein H, Semkova I, Addicks K, Schraermeyer U (2004). Neurally selected embryonic stem cells induce tumor formation after long-term survival following engraftment into the subretinal space.. Invest Ophthalmol Vis Sci.

[66] Tai YT, Svendsen CN (2004). Stem cells as a potential treatment of neurological disorders.. Curr Opin Pharmacol.

[67] Taupin P (2006). HuCNS-SC (StemCells).. Curr Opin Mol Ther.

